# Hetero/Homo-Complexes of Sucrose Transporters May Be a Subtle Mode to Regulate Sucrose Transportation in Grape Berries

**DOI:** 10.3390/ijms222112062

**Published:** 2021-11-08

**Authors:** Yumeng Cai, Ling Yin, Jie Wang, Wenjie Dong, Han Gao, Jinxu Xu, Zhefang Deng, Wenrui Tu, Jing Yan, Qingyong Meng, Yali Zhang

**Affiliations:** 1 College of Food Science and Nutritional Engineering, China Agricultural University, Beijing 100083, China; caiyumeng917@163.com (Y.C.); cauwangjie2021@163.com (J.W.); dongwenjie1134@163.com (W.D.); fadaiclub@126.com (H.G.); xujinxujason@gmail.com (J.X.); 531128763@163.com (Z.D.); twr6637@163.com (W.T.); yanjingjing1618@163.com (J.Y.); 2 Tianjin Key Laboratory of Crop Genetics and Breeding, Crops Research Institute, Tianjin Academy of Agricultural Sciences, Tianjin 300384, China; 3 Guangxi Crop Genetic Improvement and Biotechnology Key Lab, Guangxi Academy of Agricultural Sciences, Nanning 530007, China; yinling@gxaas.net; 4 The State Key Laboratory for Agrobiotechnology, College of Biological Sciences, China Agricultural University, Beijing 100193, China; qymeng@cau.edu.cn

**Keywords:** different varieties, *Vitis*, sucrose transporter, functional variation, oligomerization, functional regulation

## Abstract

The sugar distribution mechanism in fruits has been the focus of research worldwide; however, it remains unclear. In order to elucidate the relevant mechanisms in grape berries, the expression, localization, function, and regulation of three sucrose transporters were studied in three representative *Vitis* varieties. Both *SUC11* and *SUC12* expression levels were positively correlated with sugar accumulation in grape berries, whereas *SUC27* showed a negative relationship. The alignment analysis and sucrose transport ability of isolated SUCs were determined to reflect coding region variations among *V. vinifera*, *V. amurensis* Ruper, and *V. riparia*, indicating that functional variation existed in one SUT from different varieties. Furthermore, potentially oligomerized abilities of VvSUCs colocalized in the sieve elements of the phloem as plasma membrane proteins were verified. The effects of oligomerization on transport properties were characterized in yeast. VvSUC11 and VvSUC12 are high-affinity/low-capacity types of SUTs that stimulate each other by upregulating *V_max_* and *K_m_*, inhibiting sucrose transport, and downregulating the *K_m_* of VvSUC27. Thus, changes in the distribution of different SUTs in the same cell govern functional regulation. The activation and inhibition of sucrose transport could be achieved in different stages and tissues of grape development to achieve an effective distribution of sugar.

## 1. Introduction

Grapes, as non-climacteric fruits, have a double sigmoidal growth curve that is generally divided into three stages [1]. During the inception of ripening in grape berries, known as veraison, which usually occurs within 24 h, soluble sugars increase and organic acidity declines, inducing a rapid decrease in the acid/sugar balance [2]. Hence, sugar transportation is a prominent part of veraison. Sucrose is the main form of photosynthate, and principal sugars are transported through the vascular system to sink organs in higher plants [3]. Therefore, sucrose transporters (SUTs or SUCs) play a pivotal role in the translocation of sucrose during plant development.

Phylogenetic analysis has shown that these sucrose transporter proteins fall into five independent phylogenetic clades, that is, SUT1 to SUT5 [4]. Among the five clades, SUT3 and SUT5 are monocot-specific, whereas SUT1 is dicot-specific. However, SUT2 and SUT4 affect both monocots and dicots. Most members of the SUT1 clade, such as AmSUT1, function in loading the phloem or importing sucrose into sink tissues, and are localized in the plasma membranes of sieve elements (SEs) [5,6,7] or companion cells (CCs) [8], or in both cells types [9]. SUT2 clade members, such as ZmSUT2, are not only localized in SE plasma [6,7,10,11] or vacuolar membranes [12], but also function as low-affinity sugar sensors, such as AtSUT2 [13]. Additionally, some members of the SUT4 clade are responsible for sucrose/H^+^ symporters for the vacuolar storage of sucrose, and are located in vacuoles [14,15]. Other members of the SUT4 clade, such as StSUT4, are located at the plasma membrane and affect flowering, tuberization, and ethylene production [16,17]. Additionally, there are few reports regarding monocot-specific SUT3 and SUT5 clades. For wheat, TaSUT1 has been reported to be localized and function at the plasma membrane of phloem SEs for retrieving sucrose leaked to the apoplasmic phloem [18]. In *Oryza sativa*, OsSUT1 has been reported to play a role in the phloem loading of sucrose into the CCs and SEs of vascular bundles [19,20,21]. As a division from the SUT1 clade, OsSUT5 falls into the monocot-specific SUT5 clade. It shows a higher affinity for sucrose than OsSUT1, a lower pH dependence for activity, and broader substrate specificity [22].

Three putative SUT cDNAs were first cloned from grape berries (*Vitis vinifera* L.), namely *VvSUC11* (AF021808), *VvSUC12* (AF021809), and *VvSUC27* (AF021810) [23,24]. Both VvSUC11 and VvSUC12 (same as above), expressed in an invertase-deficient yeast strain, have been identified as high-affinity/low-capacity (HALC) systems (with a transporter at an optimum pH of 4.5 and a *K_m_* of 0.9 ± 1.4 mM) [25], whereas VvSUC27, extracted from *Vitis vinifera* cv. Cabernet Sauvignon, has been characterized as a low-affinity/high-capacity (LAHC) system (at an optimum pH of 4.0–5.0 and a *K_m_* of 8.0–10.5 mM) [26]. SUT expression has been reported to be involved in sugar accumulation [6,27]. Since grapes from different localities have distinct sugar concentrations, we identified that the expression of SUTs is correlated with sugar accumulation in a previous study [28]. Moreover, the differences in sugar content and other physiological responses may be due to different transporter activities among different species, in addition to expression.

However, comparisons of the amino acid sequences of one SUT from different species and the influence of amino acid differences on transporter activities remain unclear. In this study, the amino acid sequences of SUC11, SUC12, and SUC27 from *V. vinifera*, “Thompson Seedless”, *V. vinifera*, “Chardonnay”, *V. amurensis* Ruper, “Zuoshan-1”, and *V. riparia*, “DVIT1848” were isolated, and their sucrose transport abilities were determined. We further analyzed the tissue localization, interaction, and the effect of oligomerization on transport properties of grape SUTs to systematically evaluate their functions.

## 2. Results

### 2.1. SUT Expression Correlates with Sugar Accumulation

**Figure 1:**
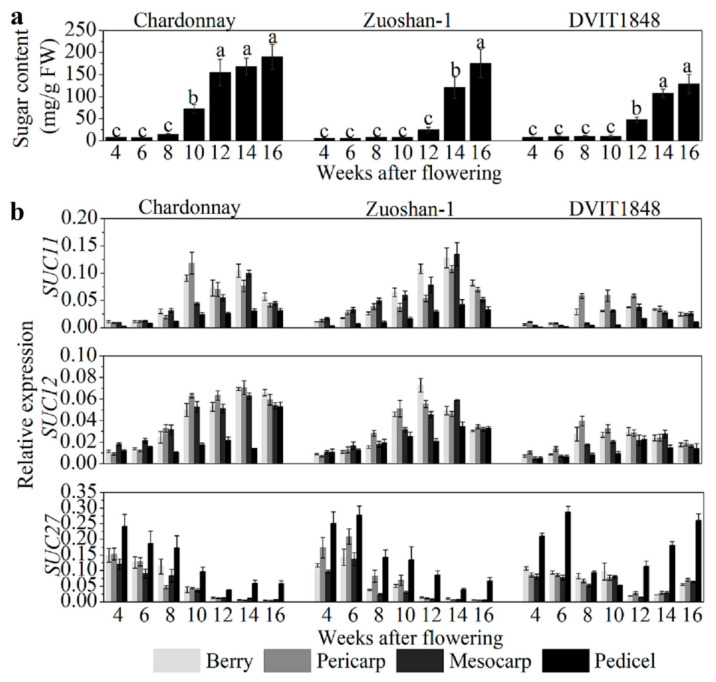
Sugar content and SUT gene expression level analysis in berries from different grapevines. (**a**) Sugar content analysis of Chardonnay, Zuoshan-1, and DVIT1848 grape varieties. Different letters indicate significant differences (*p* < 0.05) among different developing periods within one grapevine, and were determined by one-way analysis of variance (ANOVA) and Tukey’s test. (**b**) Relative expression of *SUC11*, *SUC12*, and *SUC27* in the berry, pericarp, mesocarp, and pedicel of Chardonnay, Zuoshan-1, and DVIT1848 grape varieties, respectively. Gene transcript levels were normalized against reference genes (*EF-1α* and *Actin*). The error bar indicated mean ± SD (*n* = 3).

In order to study the relationships between sugar content and SUT expression in different parts of wine berries from distinct grapevines, sugar content was determined to be relatively low in the beginning of berry development and increased after the onset of ripening (10 weeks after flowering (WAF) in Chardonnay, 14 WAF in Zuoshan-1, and 12 WAF in DVIT1848; Figure 1a). The highest sugar content measured in Chardonnay, Zuoshan-1, and DVIT1848 berries is 190.41, 175.57, and 129.20 mg/g, respectively (Figure 1a). SUT gene expression was investigated by isolating total RNA from grape berries, pericarps, mesocarps, and pedicels from different varieties. Among the SUTs, *SUC11* and *SUC12* were both expressed at similar levels in pericarps and mesocarps during the development of grape berries, but not in pedicels (Figure 1b). In general, *SUC11* and *SUC12* had a relatively higher expression in the pericarps and mesocarps than in the pedicels. The sweetest variety was Chardonnay, which showed a clearer difference in SUT expression compared to the other two varieties, particularly when compared to DVIT1848. The weakest expression of these two genes was observed at an early stage of fruit ripening [29]. In the different grape varieties, the transcript levels of *SUC11* and *SUC12* were lowest in DVIT1848, which might indicate low expression levels relative to the low-sugar-content varieties. The *SUC11* and *SUC12* expression levels were positively correlated with sugar accumulation in all organs tested in these grape varieties, except for *SUC11* in Zuoshan-1 and *SUC12* in DVIT1848 pedicels, which were slightly negatively correlated with sugar accumulation (Figure S1). Chardonnay presented relatively higher Pearson’s r values for both *SUC11* and *SUC12*, particularly in the mesocarp, which indicated that they were strongly positively correlated with sugar accumulation. Previously, we found that *SUC27* expression in the whole berry was negatively correlated with sugar accumulation [28]. Here, we measured the sugar concentration again and compared it with *SUC27* expression in separate grape berry tissues in the three grapevine varieties. *SUC27* had the highest expression in vegetative organs and was strongly expressed in the pedicels (Figure 1b). We measured a high *SUC27* expression level during the early stage of fruit ripening in all varieties, but it was downregulated after the onset of ripening in both Chardonnay and Zuoshan-1. However, *SUC27* was upregulated only during the final berry ripening stage in DVIT1848.

This downregulation resulted in a negative correlation between *SUC27* expression and sugar accumulation in the parts of all grape varieties, with the exception of DVIT1848 pedicels (Figure S1). The negative correlation with sugar content was more significant in the pericarps and mescocarps of Chardonnay berries than in Zuoshan-1 berries. This correlation pattern was consistent with the sugar content in the three grapevines. SUT protein levels in pericarps were also determined by immunolocalization, using specific antisera against peptides for each SUT, which were detected by Western blotting to prove the specificity (Figure S2) and were similar to the *SUT* expression levels (Figure S3).

### 2.2. Isolation, Alignment Analysis, and Sucrose Transport Ability of SUTs between Different Grape Varieties

**Figure 2:**
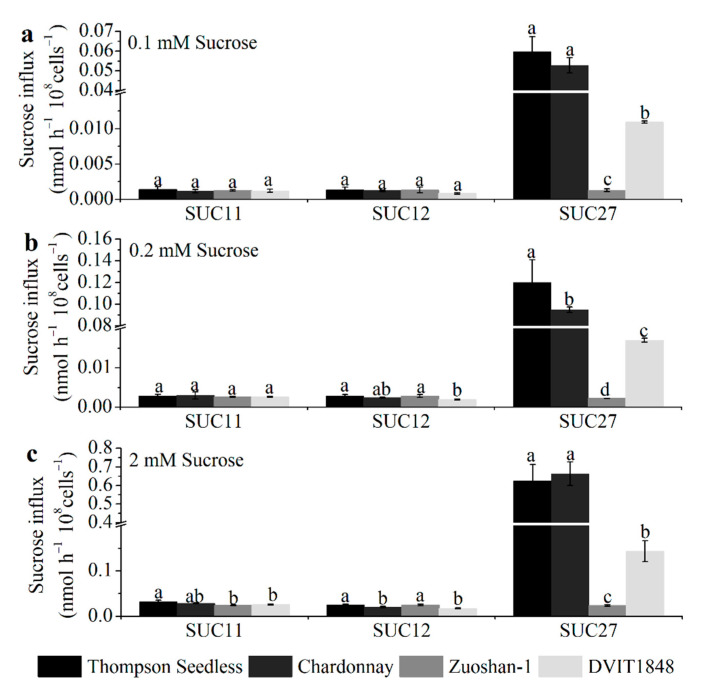
The sucrose transport ability of each SUT from Thompson Seedless, Chardonnay, Zuoshan-1, and DVIT1848, respectively. The sucrose transport ability was tested under 0.1 (**a**), 0.2 (**b**), and 2 mM (**c**) sucrose concentrations. The error bar indicated mean ± SD (*n* = 3). Different lowercase letters showed statistically significant differences among different grapevines.

To analyze the open reading frame of *SUC11* (GenBank accession no.: AF021808.1), *SUC12* (GenBank accession no.: AF021809.1), and *SUC27* (GenBank accession no.: AF021810.1) from Thompson Seedless, Chardonnay, Zuoshan-1, and DVIT1848, the total RNA of grapevine leaves was analyzed by PCR and RT-qPCR. The encoded SUC11, SUC12, and SUC27 have 500, 611, and 504 amino acids, respectively, in all three varieties, except for SUC12 in Chardonnay, which has 605 amino acids (Figure S4). Then, we simulated the models of SUTs in the four varieties using Phyre2. According to the protein data bank results, we used Yasara to compare the structural differences of each SUT type between different varieties. Overall, the models of each SUT type between the four varieties were similar (Figure S5). These differences might cause some structural modifications, such as random coils, α-helices, and β-sheets, whereas the structure, location, and number of 12 proposed core membrane-spanning regions would not change in each SUT type from different varieties. The differences in the sucrose transport ability of each SUT from different grapevines were further investigated by expressing them in the SUSY7/ura3 yeast strain on 0.1, 0.2, and 2 mM sucrose mediums (Figure 2).

Concretely, for SUC11, the amino acid sequences from the Thompson Seedless and Chardonnay varieties had only one different site between the first and second proposed core membrane-spanning regions (Figure S4a), which did not affect the structure (Figure S5a) or sucrose transport ability in any of the sucrose concentration mediums (Figure 2). The different sites for Zuoshan-1 and DVIT1848 were in the eighth and tenth proposed core membrane-spanning region, respectively (Figure S4a), which might influence the structure (Figure S5a), and significantly altered sucrose transport ability under the high-sucrose-concentration (2 mM) medium (Figure 2c) compared with Thompson Seedless.

For SUC12, the amino acid sequence from Thompson Seedless was the same as that of Zuoshan-1, whereas it lacked six amino acids at the end of the C-terminus from Chardonnay (Figure S4b), which led to some structural changes (Figure S5b) as well as a significant alteration in sucrose transport ability under the high-sucrose-concentration (2 mM) medium (Figure 2c). In terms of the amino acid sequence from DVIT1848, the different sites were in the second core membrane-spanning region and between the sixth and seventh proposed core membrane-spanning regions (Figure S4b), which resulted in structural changes (Figure S5b) and a significant alteration in sucrose transport ability under both the 0.2 mM and 2 mM sucrose concentration mediums compared with that of Thompson Seedless (Figure 2b,c), whereas the changes did not affect the sucrose transport ability between DVIT1848 and Chardonnay (Figure 2).

In terms of SUC27, the amino acid sequences from the Thompson Seedless and Chardonnay varieties had two different sites, one at the fifth proposed core membrane-spanning region and the other between the sixth and seventh proposed core membrane-spanning region (Figure S4c), which might influence the structure (Figure S5c) and alter sucrose transport ability under the 0.2 mM sucrose concentration medium (Figure 2b). Compared with *V. vinifera* varieties, the amino acid sequence from Zuoshan-1 and DVIT1848 had many different sites at and between proposed core membrane-spanning regions, respectively, which led to structural changes (Figure S5c). Furthermore, sucrose transport ability significantly decreased under every type of sucrose concentration medium, particularly for Zuoshan-1 (Figure 2).

### 2.3. Colocalization of Three SUTs in the Central Carpellary Bundle in Grape Berries

**Figure 3:**
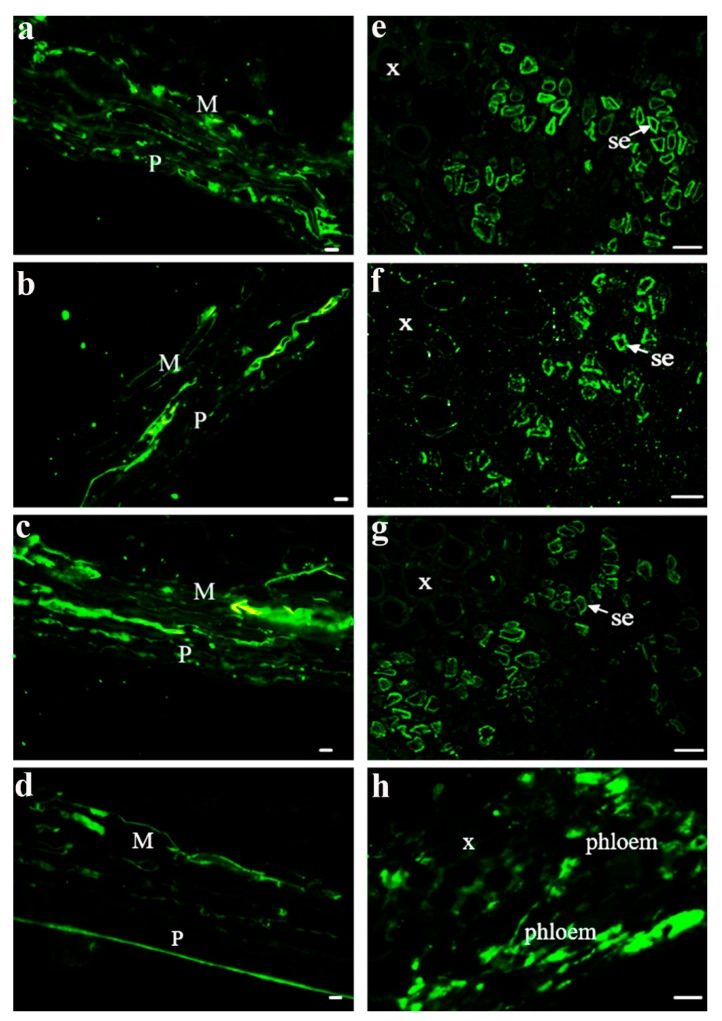
Immunolocalization of VvSUC11, VvSUC12, and VvSUC27 in the Chardonnay berry. (**a**–**d**): pericarp and mesocarp cell immunolocalization of VvSUC11, VvSUC12, VvSUC27, and β-tubulin in the Chardonnay berry. P: pericarp; M: mesocarp. Bars = 10 μm. (**e**–**h**): central vascular bundle cell immunolocalization of VvSUC11, VvSUC12, VvSUC27, and β-tubulin in the Chardonnay berry. x: xylem; se: sieve element. Bars = 10 μm.

After investigating the properties of one SUT from different grape varieties, three SUTs from *V. vinifera* were also explored. The main phloem-loading SUTs in solanaceous plants were localized at the plasma membrane in SEs by immunolocalization and transmission electron microscopy [30,31]. Here, we further investigated SUT localization in *Vitis* using the immunolocalization of three SUTs and their properties [24,25,26] to determine whether the SUTs were localized in the same tissues and/or cell types. To determine where the SUTs were localized in the berry tissues, we prepared consecutive transverse paraffin sections at veraison from Chardonnay berries to conduct fluorescent immunodetection using specific antisera against peptides in each SUT (Figure 3). Our imaging analysis showed that these three SUTs can be detected in the pericarp and mesocarp (Figure 3b,c) as well as in the SEs of the phloem from the central carpellary bundle (Figure 3e–g). β-tubulin was used as a positive control (Figure 3d,h). Combined with the results presented in Figure 1, the expression level of *VvSUC27* was significantly higher than that of *VvSUC11* and *VvSUC12* in pedicels, which are rich in conducting tissues. This high expression did not change during fruit development. VvSUC27 was located in the SEs of the conducting tissue (Figure 3), and combined with its LAHC characteristic, we speculated that VvSUC27 might maintain a high speed of sucrose transport, whereas VvSUC11 and VvSUC12 might play a role in ancillary regulation in the phloem.

**Figure 4:**
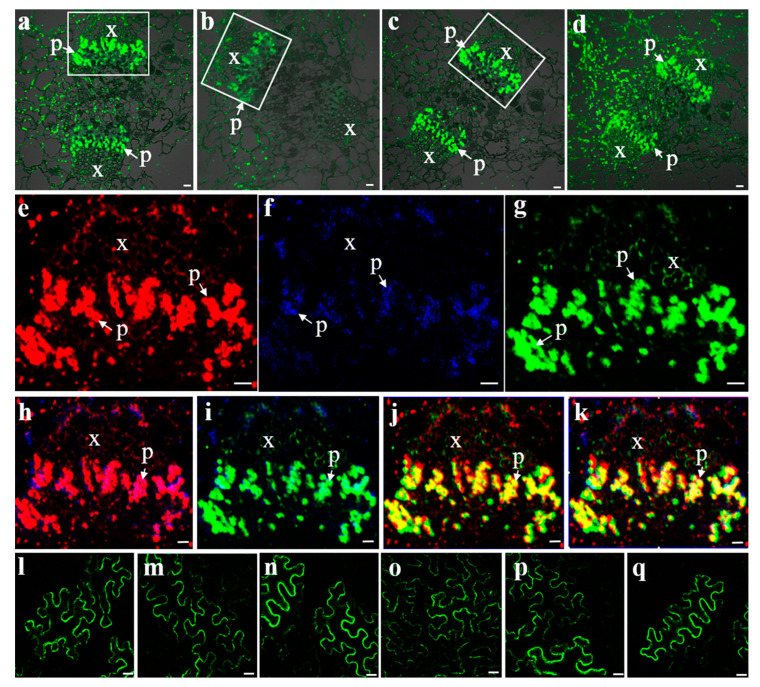
Fluorescent co-immunolocalization and BiFC interactions of VvSUCs. Fluorescent immunolocalization of VvSUC11 (**a**), VvSUC12 (**b**), VvSUC27 (**c**), and β-tubulin (**d**) in the central carpellary bundle of grapes. (**e**–**g**) Fluorescent co-immunolocalization of VvSUCs in the central carpellary bundle of grapes were marked by white boxes in (**a**–**c**). (**e**) Highlighted central carpellary bundle fluorescent immunolocalization of VvSUC11 in red. (**f**) Highlighted central carpellary bundle fluorescent immunolocalization of VvSUC12 in blue. (**g**) Highlighted central carpellary bundle fluorescent immunolocalization of VvSUC27 in green. (**h**) Fluorescent co-immunolocalization of VvSUC11 and VvSUC12. (**i**) Fluorescent co-immunolocalization of VvSUC12 and VvSUC27. (**j**) Fluorescent co-immunolocalization of VvSUC12 and VvSUC27. (**k**) Fluorescent co-immunolocalization of VvSUC11, VvSUC12, and VvSUC27. x: xylem; p: phloem. Scale bar, 10 μm. (**l**–**q**): BiFC interactions between two homo- or hetero-SUTs. For homodimers, (**l**) YFP-VvSUC11 and VvSUC11-YFP, (**m**) YFP-VvSUC12 and VvSUC12-YFP, and (**n**) YFP-VvSUC27 and VvSUC27-YFP were co-expressed in tobacco. For hetero-oligomers, (**o**) YFP-VvSUC11 and VvSUC12-YFP, (**p**) YFP-VvSUC12 and VvSUC27-YFP, and (**q**) YFP-VvSUC27 and VvSUC11-YFP were co-expressed in tobacco. Scale bar, 10 μm.

Three SUTs were detected by strong fluorescence signals in the phloem of the central carpellary bundles (Figure 4a–c). The same sections of these bundles in the image were removed and the fluorescence for VvSUC11, VvSUC12, and VvSUC27 were pseudo-colored as red, blue, and green, respectively, to evaluate the colocalization of the three SUTs within the same tissue (Figure 4e–k). VvSUC11, VvSUC12, and VvSUC27 could be colocalized in the same SE cell; therefore, we asked whether the three SUTs could interact with each other. To determine whether the three SUTs in grapevines form homo-oligomerized and hetero-oligomerized complexes in plant cells, a BiFC assay was performed. Expression vectors for VvSUC11 (or VvSUC12/VvSUC27) from the leaves of Thompson Seedless fused to the N-terminal or C-terminal half of YFP were constructed and co-transformed into *N. benthamiana* by measuring the fluorescence of reconstituted YFP, and we found that VvSUC11, VvSUC12, and VvSUC27 can form homodimers (Figure 4l–n) and hetero-oligomers (Figure 4o–q).

### 2.4. The Potential Effect of SUT Oligomerization on the Transport Properties of Yeast

**Figure 5:**
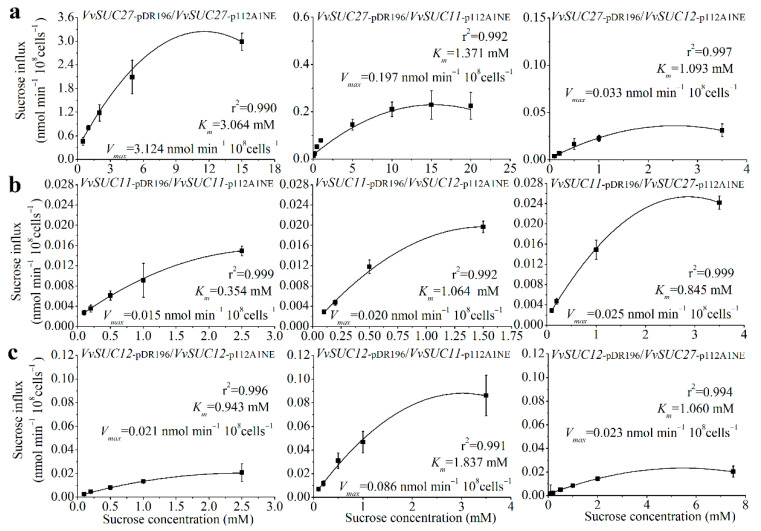
Sucrose uptake into yeast cells (SUSY7/ura3) was measured after co-expressing different *VvSUC*s. (**a**) *VvSUC27* under the strong *PMA1* promoter (pDR196) was co-expressed with each *VvSUC* driven by the weaker *ADH1* promoter (p112A1NE). (**b**) *VvSUC11* under the strong *PMA1* promoter was co-expressed with each *VvSUC* driven by the weaker *ADH1* promoter. (**c**) *VvSUC12* under the strong *PMA1* promoter was co-expressed with each *VvSUC* driven by the weaker *ADH1* promoter. The error bar indicated mean ± SD (*n* = 3).

Membrane proteins are capable of forming oligomeric structures that regulate transport [30,31]. To determine how oligomerization affects sucrose transport properties of the SUTs in *Vitis*, the three SUTs from the leaves of Thompson Seedless were co-expressed in the SUSY7/ura3 yeast strain (Figure 5). First, the values of *V_max_* and the sucrose affinity (*K_m_*) of VvSUC27 were nearly 200-fold and 150-fold higher than those of VvSUC11 and VvSUC12, respectively, whereas the *V_max_* and *K_m_* values of VvSUC11 and VvSUC12 were similar. These findings are consistent with previous reports [24,25,26]. VvSUC27 has the lowest sucrose affinity; therefore, it was expressed under the strong *PMA1* promoter in the pDR196 vector and co-expressed with *VvSUC11* driven by the weaker *ADH1* promoter in the p112A1NE vector. This co-expression induced a 2.23-fold increase in affinity and a 13.64-fold decrease in *V_max_*. When *VvSUC27* in pDR196 was co-expressed with *VvSUC12* in p112A1NE, there was a 2.80-fold increase in sucrose affinity and a 94.67-fold reduction in *V_max_* (Figure 5a). *VvSUC11* was cloned into the pDR196 vector, which reduced sucrose affinity (*K_m_*) by 3.01-fold when co-expressed with *VvSUC12* in the p112A1NE vector, and a 1.33-fold increase in *V_max_* was observed (Figure 5b). However, when *VvSUC27* was cloned in the p112A1NE vector and co-expressed with *VvSUC11* under a strong promoter in the pDR196 vector, sucrose affinity decreased by 2.39-fold and *V_max_* increased by 1.67-fold. When *VvSUC12* was expressed under the strong *PMA1* promoter, a 1.95-fold reduction in affinity and a 4.10-fold increase in *V_max_* were observed when it was co-expressed with *VvSUC11* driven by the weaker *ADH1* promoter (Figure 5c). A 1.12-fold decrease in affinity was observed when *VvSUC12* was co-expressed with *VvSUC27* in p112A1NE, as was a 1.10-fold reduction in *V_max_*. These results indicate that both VvSUC11 and VvSUC12 could inhibit the sucrose transport ability of VvSUC27, especially VvSUC12. VvSUC11 and VvSUC12 interact and activate each other. After activation, we observed a 1.33- to 4.10-fold increase in *V_max_* and a 3.01- to 1.95-fold upregulation in *K_m_*. Considering the high values of *V_max_* and *K_m_* of VvSUC27 itself, whether VvSUC27 could induce the sucrose transport ability of VvSUC11 and VvSUC12 could not be determined, since there was no obvious induction.

## 3. Discussion

### 3.1. The Diverse Expression Patterns of SUTs Are Related to Fruit Sugar Contents in Different Vitis Species

The sugar content of grapes is linked to wine quality; grapes grown in different locations have distinct properties and sugar concentrations, and the transmembrane transport of sucrose requires the activity of SUTs. Various studies on SUTs in higher plants have been reported. However, there are limited reports on *Vitis*. At least 60 *Vitis* species have been isolated worldwide, and among them the European wine grape (*V. vinifera*) accounts for the majority of global wine production, even though it has a high susceptibility to diseases and a low cold tolerance [32]. Amur grapes (*V. amurensis* Rupr.) are important wild fruit crops with strong biotic and abiotic stress resistance, which has elicited interest in the wine production industry [33]. Native American species (e.g., *V. riparia*) also have better resistance than *V. vinifera*, and as a result are popular in wine-producing areas with continental and humid climates [32]. In our study, we chose Chardonnay, Zuoshan-1, and DVIT1848, which are representative wine-grape species from different areas, in order to investigate the relationship between sugar content and SUTs.

Among these three grape varieties, Chardonnay berries had the highest sugar content, Zuoshan-1 had a medium content, and DVIT1848 had the lowest. The sugar content differences depended on the expression patterns of SUTs among the different varieties (Figure 1). Overall, *SUC11* and *SUC12* accumulated at the onset of ripening and then more significantly at, or after, veraison in grape berries and tissues in all varieties, which was similar to previous reports [23,29]. These expression patterns suggest that *SUC11* and *SUC12* are essential for sugar accumulation during the later stages of berry development. Conversely, *SUC27* was most expressed at the early stages of berry development [28]; when phloem unloading shifted from the symplasmic to the apoplasmic pathway, the expression of *SUC27* was downregulated concomitantly with an apparent block in a small portion of plasmodesmata and phloem strands during the ripening and late mature stages, respectively [34]. We speculate that *SUC27* might be responsible for phloem loading, which is supported by the high expression of *SUC27* measured in pedicels and verified by their SE location. Furthermore, the SUTs were expressed at the highest levels in Chardonnay, then in Zuoshan-1, and lowest in DVIT1848, consistent with the differences in sugar content between these species. Therefore, higher sugar contents depended on the degree of positive correlation with *SUC11* and *SUC12* expression and a negative correlation with *SUC27* expression in grape berries and berry tissues (Figure S1). Our results suggest that *SUT* expression levels may cause differences in sugar content among different grape varieties.

### 3.2. Conservation and Divergence of SUTs from Different Vitis Species

A comprehensive understanding of the molecular differences in one gene in different species is also a key step towards understanding their physiological roles in different growth phases. Specific amino acid variations were discovered through the alignment of the allelic haplotypes from each sugarcane SUT, indicating that potential functional variation existed among these allelic haplotypes [35]. However, how the differences in sequences of amino acids among different species affect the SUT function remains unclear. In this study, the comparative analyses of each SUT from different *Vitis* species showed that amino acids in the SUC11 and SUC12 groups were more conservative than those in the SUC27 group (Figure S4), and further provided direct alterations in structure (Figure S5) and sucrose transport ability (Figure 2). Furthermore, evolution of the gene structure after these plant divergences did not cause significant variations in the coding region in the two *V. vinifera* varieties, whereas some variations in the coding region occurred among *V. vinifera*, *V. amurensis* Ruper, and *V. riparia* varieties (Figure S4), which were reflected in the significant changes in sucrose transport ability, especially under the high-sucrose-concentration medium (Figure 2), indicating that functional variation existed among one gene from different varieties.

### 3.3. VvSUC11 and VvSUC12 Regulate SUT-Dependent Sucrose Transport and Have Limited Sucrose Transport Capacity

Three SUTs from *V. vinifera* were investigated. It has been reported that three SUTs (SUT1, SUT2, and SUT4) with different properties were detected in the same SEs of tissues in potatoes [30]. Here, VvSUC11 and VvSUC12 were also localized in the plasma membrane of phloem SEs in *V. vinifera* (Figure 3).

It is worth noting that VvSUC11 has been reported to have a high affinity for sucrose [24,25], which has been verified in this study (Figure 5). Among the SUT4 clade, only DcSUT1 in carrots was reported to be a high-affinity SUT, expressed only in the green parts of plants, with the highest levels in the lamina of source leaves [36]. VvSUC11 has also been reported to be highly expressed in mature leaves (source leaves) and stems [29], suggesting that VvSUC11 may be required for loading sucrose into the phloem. SUT2 in tomatoes and Arabidopsis lacks detectable transport activity [7], whereas PmSUC3 transports ^14^C-labeled sucrose across the plasma membrane with a low affinity in PmSUC3-expressing IBY20 yeast (the *K_m_* for sucrose is 5.5 mM) [10]. VvSUC12 has also been reported to have a relatively high affinity for sucrose [24,25], which is consistent with our findings (Figure 5). In tomatoes, LeSUT2 co-localizes with high- and low-affinity LeSUT1 and LeSUT4 [7]. Both VvSUC11 and VvSUC12 colocalize with each other and VvSUC27 in SE cells (Figure 4), suggesting that VvSUC11 and VvSUC12 can form homo-oligomers and hetero-oligomers (Figure 4). The binding of the substrate on the outside or inside of one subunit could affect the activity status of the complex through co-operative conformational changes [37]. VvSUC11 and VvSUC12 could activate each other or inhibit the sucrose transport ability of VvSUC27 (Figure 5).

### 3.4. VvSUC27 Has Strong Sucrose Transport Capacity but Could Be Regulated by Other SUTs

VvSUC27 belongs to the specific high-affinity SUT1 clade in dicots [29]. Here, VvSUC27 was identified and colocalized with VvSUC11 and VvSUC12 at the plasma membrane in SEs (Figure 3 and Figure 4). Our previous study showed that *VvSUC27* was highly expressed in several vegetative organs, including mature leaves [38]. Moreover, VvSUC27 is characterized as a LAHC SUT with a sucrose uptake activity that is activated by monosaccharides and inhibited by maltose and diethyl pyrocarbonate [26]. The capacity of VvSUC27 to form hetero-oligomers with both VvSUC11 and VvSUC12 decreased as sucrose affinity increased (Figure 5), indicating that the function of VvSUC27 may be different from that of the other two SUTs.

It has been reported that the phloem unloading of sucrose shifts from the symplasmic to the apoplasmic pathway during grape berry development [34]. A symplasmic unloading pathway predominates in the early and middle stages, while an apoplasmic unloading pathway operates in the ripening stage. This shift is involved in the onset of ripening in grape berries and a small portion of plasmodesmata were apparently blocked after the shift [34] and our results showed that VvSUCs were also affected by this shift (Figure 1). The pH of apoplasmic space is around 5, while the pH of cytosol is around 7. Since SUT family members are H+/sucrose symporters, they are energized by proton pump ATPase [8]. Before the onset of ripening, VvSUC27 acted predominately in SE as an LAHC SUT, which might be essential for maintaining the sucrose gradient and symplasmic transport, whereas the proportion of VvSUC27 is dozens or even hundreds of times that of VvSUC11 or VvSUC12. At this time, VvSUC27 may mostly exist on the cell membrane in the form of homologous dimers or polymers, and the co-executor guided the rapid transportation of sucrose in the SE. After the onset of ripening, there was little variation in the expression of the three SUTs in SE, except for the blocking or functional inhibition of plasmodesma, which likely accounted for the shift to the apoplasmic pathway [34]. During this stage, the expression pattern of the three SUTs reversed in phloem cells, the expression of VvSUC27 began to decrease, the expression of VvSUC11 and VvSUC12 began to increase, and the ratio of VvSUC27 to VvSUC11 and VvSUC12 decreased. Meanwhile, VvSUC27 formed a heterodimer or polymer with VvSUC11 or VvSUC12, resulting in a significant reduction in the ability of VvSUC27 to transport sucrose. The ability of VvSUC27 was inhibited because of the higher populations of VvSUC11 and VvSUC12. This proportion of SUC transporters is similar to what we observed in yeast when VvSUC27 was expressed in pDR196 and co-expressed with VvSUC12 or VvSUC12 in 112A1NE (Figure 5). In the mesocarp, the expression level of VvSUC27 was dozens of times higher than that of VvSUC11 and VvSUC12 before veraison. Although the proportion of VvSUC27 was dominant, its function might be inhibited; at the beginning of the ripening stage, the expression of VvSUC27 decreased sharply, while VvSUC11 and VvSUC12 increased rapidly and became the dominant position, which was dozens of times higher than that of VvSUC27. Although VvSUC11 and VvSUC12 are HALC SUTs, they can activate each other, raise their *K_m_* and sucrose transport abilities, and control the transportation of sucrose in mesocarp cells.

In summary, the three SUTs from grapes often co-exist in cells, and they can form homologous or heterologous dimers or polymers to regulate the transport capacity of sucrose. There are two types of regulation: activation and inhibition, which can be realized in different stages in the development and tissues of grape berries to achieve an effective distribution of sucrose.

## 4. Materials and Methods

### 4.1. Plant Material

Grapevine berries and leaves of 3-year-old *V. vinifera*, “Thompson Seedless”, *V. vinifera*, “Chardonnay”, *V. amurensis* Ruper, “Zuoshan-1”, and *V. riparia*, “DVIT1848” were collected in the growing season (between 10 May and 15 June) from Shangzhuang (Beijing, China). The berries and leaves were sampled, immediately frozen in liquid nitrogen, and stored at −80 °C.

### 4.2. Soluble Sugar Measurement

For the extraction and measurement of soluble sugar, frozen grape berries (5 g) were ground, dissolved in 30 mL of ddH_2_O, and then distilled three times at 100 °C for 30 min. The extract solutions were diluted with ddH_2_O to a final volume of 100 mL. Measurements were performed using an anthrone-sulfuric acid colorimetric assay, as previously described in [39].

### 4.3. Generation of Anti-SUT Antisera and the Immunolocalization of SUT Proteins

Specific peptide fragments from VvSUC11 (PGGHRQRGRPR and SSADKSRVHT), VvSUC12 (VPYKNLKQAEVE and QQIGFDNSKSKLDM), and VvSUC27 (VPNTKDERTQPSS and FRQLRRPMW) were derived from the protein sequences used to synthesize polypeptides. For each protein, both peptide fragments were mixed and used for the immunization of three specific-pathogen-free rabbits (BG Biotech Co., Ltd., Beijing, China), and the polyclonal antibodies were obtained by affinity purification. The specific polyclonal antibodies for each SUT were confirmed by Western blotting using grape berries from Chardonnay [40].

For immunohistochemical analyses, berries were collected two weeks before and after veraison from Chardonnay, Zuoshan-1, and DVIT1848 plants cultivated in the same field (Shangzhuang, Beijing, China) and processed under the same conditions. The berries were embedded in paraffin and cut into transverse sections. After dewaxing, the slides were treated with horseradish-peroxidase-labeled antibodies, as previously described in [41]. The specimens were then viewed with an Axio Scope A1 microscope (Zeiss, Oberkochen, Germany).

The fluorescent immunodetection of SUTs was performed as previously described in [5]. Chardonnay berries collected at veraison were analyzed from four continuous transverse paraffin sections; three were used for SUT localization and one for tubulin, as a control. Fluorescence was observed using a Nikon C1 Si/TE2000E confocal laser scanning microscope (Tokyo, Japan), and EZ-C1 v3.00 software was used for image processing. Image J software was used to adjust the fluorescence pseudo-color and construct colocalization images. ANDOR Dragonfly (Belfast, UK) was used observe the sieve element cells.

### 4.4. RNA Extraction and RT-qPCR

Total RNA from grape berries and tissues was isolated using a HiPure Plant RNA Kit (Guangzhou, China). cDNA was synthesized using a HiFiScript gDNA Removal cDNA Synthesis Kit (CWBIO, Beijing, China). To normalize the cDNA samples, EF-1α and actin from the grapes were selected as internal controls. Gene-specific primers (Table S1) were designed, and RT-qPCR was performed using an UltraSYBR Mixture Kit (CWBIO, Beijing, China) with a Rotor-Gene^®^SYBR^®^Green PCR Kit (QIAGEN, Hilden, Germany). To determine the different concentrations of cDNA, the threshold cycle for each RT-qPCR was identified and compared against the internal standards (*EF-1α* and *actin*).

### 4.5. Isolation of SUT Amino Acid Sequences

cDNA was synthesized as previously described. The complete SUC11, SUC12, and SUC27 coding sequences were amplified from the cDNA of Thompson Seedless, Chardonnay, Zuoshan-1, and DVIT1848. The primer sequences used are listed in Table S1. Then, the sequences were cloned with pGEM-T Easy Vector (Promega, Madison, WI, USA) and subsequently sequenced.

### 4.6. Bioinformatics Software Analysis

The gene sequences were transformed into amino acid sequences using Primer Premier v5.0 software. Different types of grapevines for each SUT amino acid sequence were processed by multiple sequence alignment using DNAMAN software. The transmembrane domains were predicted using THMMH Server v2.0 (http://www.cbs.dtu.dk/services/TMHMM-2.0/ (accessed on 10 July 2018) [42,43]. The SUT model was simulated using the website http://www.sbg.bio.ic.ac.uk/phyre2/html/ (accessed on 17 February 2019). The structural alignments were processed using Pymol v1.8 software.

### 4.7. Sucrose Uptake Assay

The SUT sequences obtained from the cDNA of Thompson Seedless were chosen as templates with which to construct the expressed plasmids pDR196 and p112A1NE [30,44]. The primer sequences used are listed in Table S1. The sucrose-uptake-deficient yeast strain, SUSY7/ura3, which cannot grow efficiently on a medium containing sucrose as the sole carbon source unless a sucrose uptake system is provided through ectopic expression, was chosen as the host for ^14^C-sucrose uptake [7,26,30,45]. A yeast sucrose uptake assay was performed as previously reported, with some modifications [26,46], and the transformants were grown in a minimal medium to an optical density at *OD_623_* of 0.6, then washed twice (20 mL each time) with 25 mM of sodium phosphate buffer (pH of 5.0) and suspended in the same buffer to an optical density of 20 at *OD_623_*. The yeast cell suspension (100 μL) was mixed with glucose (pH of 5.0) to a final concentration of 10 mM for 1 min at 30 °C. As previous experiments and reports have shown [25,26], a linear rate of sucrose uptake was maintained for the first 20 min; therefore, uptake assays were initiated by immediately adding different concentration gradients of mixed ^14^C-sucrose at 30 °C in a shaking water bath for 20 min. Cells were collected by centrifugation and washed twice with 10 mM of ice-cold sucrose (25 mM of dissolved sodium phosphate buffer, pH of 7.0). The radioactivity bound to the filters was determined using liquid scintillation counting. The background to the sucrose caused by the SUSY7/ura3 yeast strain was subtracted.

### 4.8. Bimolecular Fluorescence Complementation (BiFC)

The vectors pSPYNE and pSPYCE, which have the CaMV 35S promoter, were used to drive the expression of the N- and C-terminal halves of YFP, respectively. In this study, the N-terminal half of YFP in pSPYNE was positioned before a multiple cloning site, whereas the C-terminal half of YFP was located in pSPYCE, as previously reported in [47]. The coding sequences for VvSUC11, VvSUC12, and VvSUC27 from Thompson Seedless were cloned into pSPYNE and pSPYCE using gene-specific primers (Supplemental Table S1).

Fluorescence was observed using a Nikon C1 Si/TE2000E confocal laser scanning microscope, and EZ-C1 v3.00 software was used for image processing.

### 4.9. Statistical Analyses

One-way analysis of variance (ANOVA) and Tukey’s test were performed to analyze the significance of data using SPSS v16.0 (SPSS Corp., Chicago, IL, USA). The statistical significance was set at *p* < 0.05. The relationships between SUT gene expression and sugar accumulation were explored using Pearson’s correlation coefficient. Pearson’s r was always between −1 and +1, where −1 refers to a perfect negative relationship, +1 refers to a perfect positive relationship, and 0 refers to the absence of a relationship.

## Data Availability

Not applicable.

## Supplementary Materials

The following are available online at https://www.mdpi.com/article/10.3390/ijms222112062/s1.
