# Supplementary material for: Hetero/Homo-Complexes of Sucrose Transporters May Be a Subtle Mode to Regulate Sucrose Transportation in Grape Berries
Source: Int J Mol Sci. 2021 Nov 8;22(21):12062. doi: 10.3390/ijms222112062 (PMC8584533; doi:10.3390/ijms222112062)
Supplement: Supplementary file 1 [file ijms-22-12062-s001.zip › Supplementary Figures S1-S5 and Table S1.pdf]

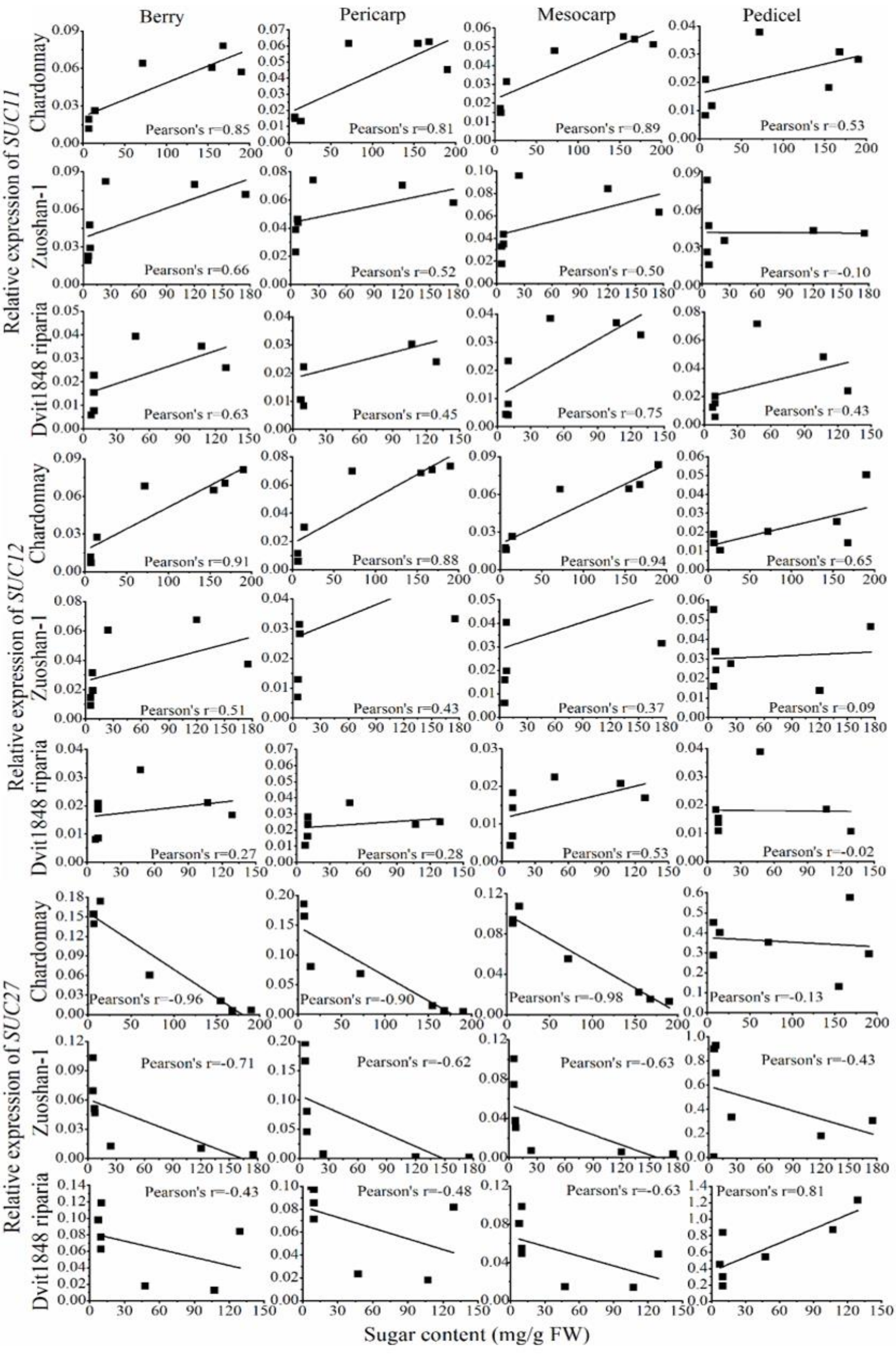

Figure S1. Pearson correlation coefficients were calculated to determine the correlation between SUT gene expression in berries, pericarps, mesocarps, pedicels and the sugar content in Chardonnay, Zuoshan-1, and DVIT1848. Data are expressed as the mean ± S.D. from three biological replicates.

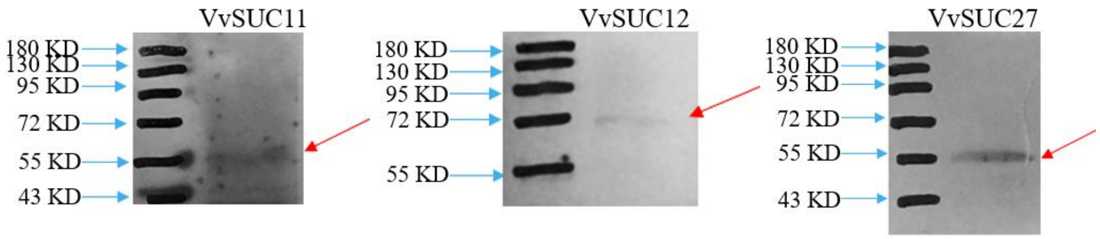

Figure S2. Certification for the specificity of specific antisera against peptides for each SUT by western blotting.

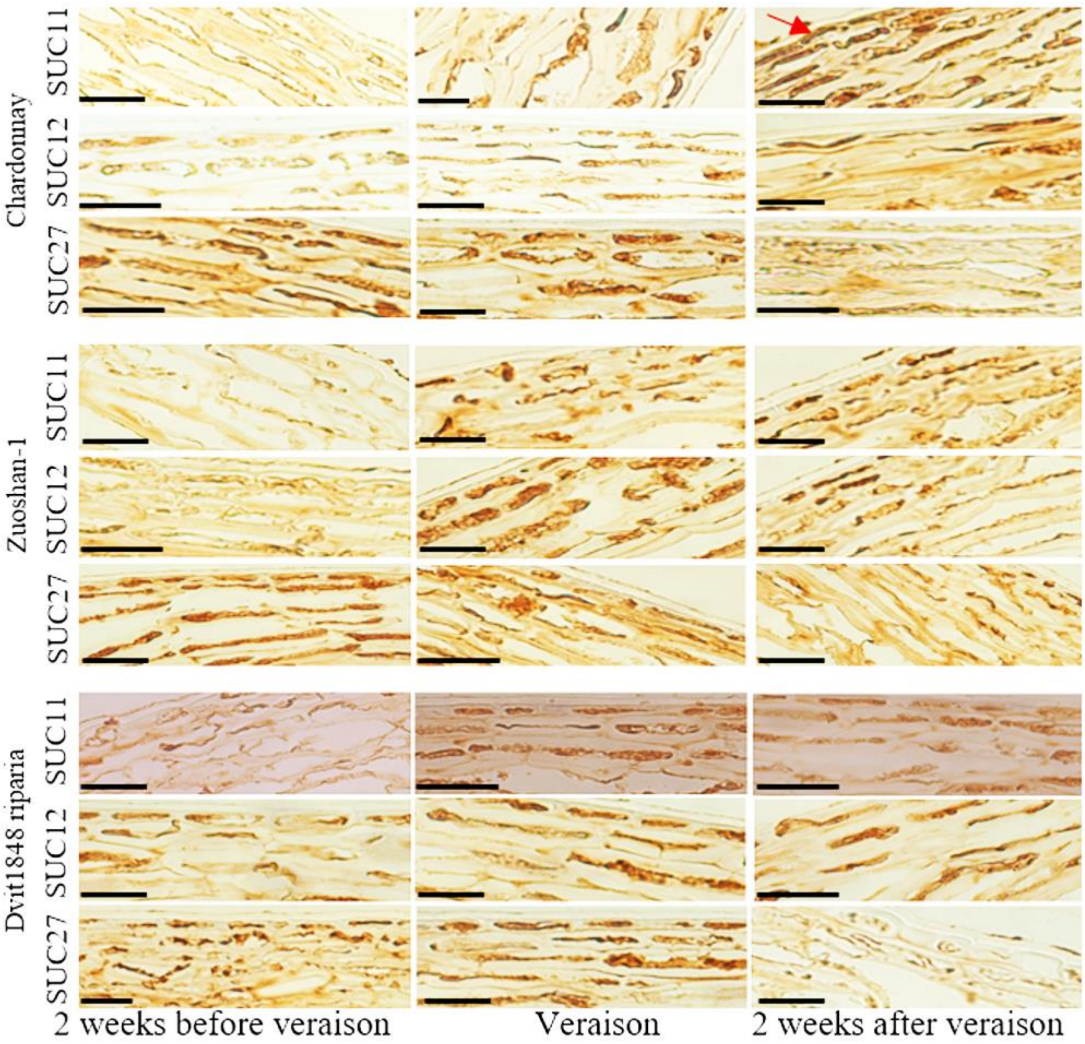

Figure S3. Immunohistochemical semi-quantitative analysis of SUC11, SUC12, and SUC27 in pericarp from Chardonnay, Zuoshan-1, and DVIT1848, respectively. Bars = 100 μm. Red arrow indicated positive points.

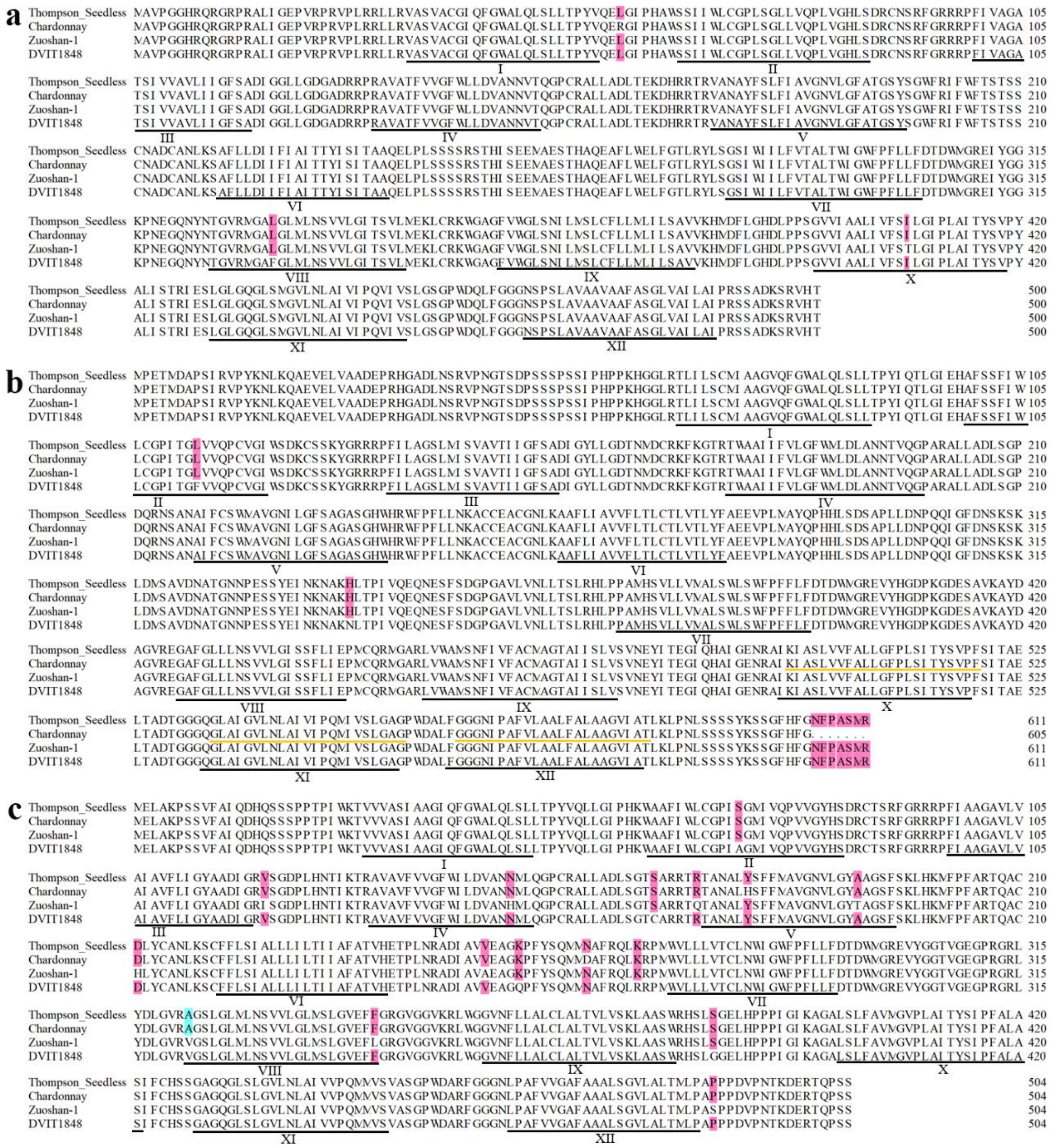

Figure S4. Alignment of the amino acid sequences of SUC11 (a), SUC12 (b), and SUC27 (c) from Thompson Seedless, Chardonnay, Zuoshan-1, and DVIT1848, respectively. Amino acid sequences were aligned using the DNAMAN program. Twelve proposed core membrane spanning regions in amino acids across all the sequences were indicated by black and yellow lines. The difference between grape varieties was marked in pink.

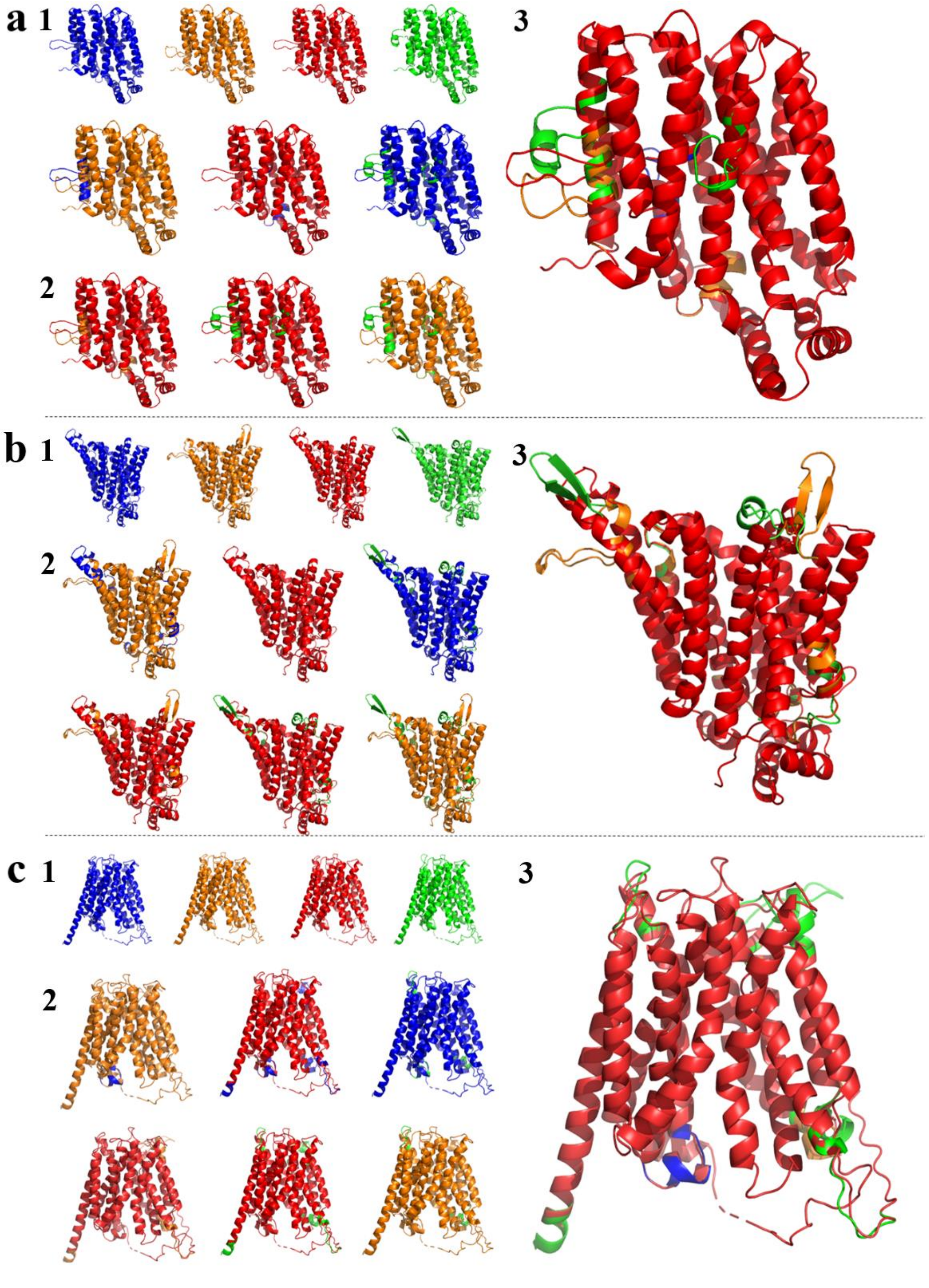

Figure S5. Alignment on the protein structure prediction of SUC11 (a), SUC12 (b), and SUC27 (c) from Thompson Seedless (blue), Chardonnay (brown), Zuoshan-1 (red), and DVIT1848 (green), respectively. For numbers, "1" represented the single structure from one grape variety, "2" represented the compared structure between two selected grape varieties, and "3" represented the compared structure among all the selected grape varieties.

Table S1. Primers used in this study.

Gene name	Primer Sequence (5'-3')
SUC11<br>(qRT-PCR)	For；GTCGCCGTTCTGATAATC<br>Rev：ATTAGCCACATCCAATAACC
SUC12<br>(qRT-PCR)	For：GCAGGCTGAGGTTGAATTG<br>Rev：GTGGATGAGGAATGGATGAAG
SUC27<br>(qRT-PCR)	For：CGTTATCTCTGTTCGCTGTTATG<br>Rev：GCTACCGACACCATCATCTG
VvEF-1a<br>(qRT-PCR)	For：GAACTGGGTGCTTGATAGGC<br>Rev：CCAAAATATCCGGAGTAAAAG
VvActin<br>(qRT-PCR)	For：AGCTGGAAACTGCAA AGAGCAG<br>Rev：ACA ACGGAATCTCTCAGCTCCA
SUC11<br>(full length sequence)	For；ATGGCGGTCCCTGGGGGCC<br>Rev；TCATGTGTGGACCCTGGATTTATC
SUC12<br>(full length sequence)	For；ATGCCGGAGACGATGGACGC<br>Rev；TCACCTCATTGAGGCAGGGAAG
SUC27<br>(full length sequence)	For；ATGGAGTTAGCCAAGCCTTC<br>Rev；TTAAGACGACGGCTGAGTCC
pSPYNE-*VvSUC11*	For：ACGCGTCGACATGGCGGTCCCTGGGGGCC<br>Rev：TCCCCCCGGGTCATGTGTGGACCCTGGATTTATC
pSPYNE-*VvSUC12*	For：ACGCGTCGACATGCCGGAGACGATGGACGC<br>Rev：TCCCCCCGGGTCACCTCATTGAGGCAGGGAAG
pSPYNE-*VvSUC27*	For：ACGCGTCGACATGGAGTTAGCCAAGCCTTC<br>Rev：TCCCCCCGGGTTAAGACGACGGCTGAGTCC
pSPYCE-*VvSUC11*	For：CTAGTCTAGAATGGCGGTCCCTGGGGGCC<br>Rev：TCCCCCCGGGTGTGTGGACCCTGGATTTATC
pSPYCE-*VvSUC12*	For：CTAGTCTAGAATGCCGGAGACGATGGACGC<br>Rev：TCCCCCCGGGCCTCATTGAGGCAGGGAAG
pSPYCE-*VvSUC27*	For：CTAGTCTAGAATGGAGTTAGCCAAGCCTTC<br>Rev：TCCCCCCGGGAGACGACGGCTGAGTCCTC
pDR196-*VvSUC11*	For：TCCCCCCGGGATGGCGGTCCCTGGGGGCC<br>Rev：ACGCGTCGACTCATGTGTGGACCCTGGATTTATC
pDR196-*VvSUC12*	For：TCCCCCCGGGATGCCGGAGACGATGGACGC<br>Rev：ACGCGTCGACTCACCTCATTGAGGCAGGGAAG
pDR196-*VvSUC27*	For：TCCCCCCGGGATGGAGTTAGCCAAGCCTTC<br>Rev：ACGCGTCGACTTAAGACGACGGCTGAGTCC
p112A1NE-*VvSUC11*	For：AAGCTTCAGCTGCTGCAGTCTAGAGAATTC<br>ATGGCGGTCCCTGGGGGCCATCGG<br>Rev：CCTCTGGCGAAGAAGTCCAAAGCTGGATCC<br>TCATGTGTGGACCCTGGATTTATC
p112A1NE-*VvSUC12*	For：AAGCTTCAGCTGCTGCAGTCTAGAGAATTC<br>ATGCCGGAGACGATGGACGC<br>Rev：CCTCTGGCGAAGAAGTCCAAAGCTGGATCC<br>TCACCTCATTGAGGCAGGGAAG
p112A1NE-*VvSUC27*	For：AAGCTTCAGCTGCTGCAGTCTAGAGAATTC<br>ATGGAGTTAGCCAAGCCTTC<br>Rev：CCTCTGGCGAAGAAGTCCAAAGCTGGATCC<br>TTAAGACGACGGCTGAGTCC
